# CYP2E1 deficit mediates cholic acid-induced malignant growth in hepatocellular carcinoma cells

**DOI:** 10.1186/s10020-024-00844-5

**Published:** 2024-06-07

**Authors:** Zhiwei Hao, Xuemin Liu, Huanhuan He, Zhixuan Wei, Xiji Shu, Jianzhi Wang, Binlian Sun, Hongyan Zhou, Jiucheng Wang, Ying Niu, Zhiyong Hu, Shaobo Hu, Yuchen Liu, Zhengqi Fu

**Affiliations:** 1 Department of Pathology and Pathophysiology, School of Medicine, Jianghan University, Wuhan, 430056 China; 2 Cancer Institute, School of Medicine, Jianghan University, Wuhan, 430056 China; 3 Hubei Key Laboratory of Cognitive and Affective Disorders, Jianghan University, Wuhan, 430056 China; 4 Wuhan Institute of Biomedical Sciences, School of Medicine, Jianghan University, Wuhan, 430056 China; 5 Liver transplant center, Union Hospital, Tongji Medical College, Huazhong University of Science and Technology, Wuhan, 430022 China; 6 Department of Pathology, Renmin Hospital of Huangpi District of Jianghan University, Wuhan, 430399 China

**Keywords:** Cholic acid, CYP2E1, Hepatocellular cancer, Cell growth, Autophagy

## Abstract

**Background:**

Increased level of serum cholic acid (CA) is often accompanied with decreased CYP2E1 expression in hepatocellular carcinoma (HCC) patients. However, the roles of CA and CYP2E1 in hepatocarcinogenesis have not been elucidated. This study aimed to investigate the roles and the underlying mechanisms of CYP2E1 and CA in HCC cell growth.

**Methods:**

The proteomic analysis of liver tumors from DEN-induced male SD rats with CA administration was used to reveal the changes of protein expression in the CA treated group. The growth of CA-treated HCC cells was examined by colony formation assays. Autophagic flux was assessed with immunofluorescence and confocal microscopy. Western blot analysis was used to examine the expression of CYP2E1, mTOR, AKT, p62, and LC3II/I. A xenograft tumor model in nude mice was used to examine the role of CYP2E1 in CA-induced hepatocellular carcinogenesis. The samples from HCC patients were used to evaluate the clinical value of CYP2E1 expression.

**Results:**

CA treatment significantly increased the growth of HCC cells and promoted xenograft tumors accompanied by a decrease of CYP2E1 expression. Further studies revealed that both in vitro and in vivo, upregulated CYP2E1 expression inhibited the growth of HCC cells, blocked autophagic flux, decreased AKT phosphorylation, and increased mTOR phosphorylation. CYP2E1 was involved in CA-activated autophagy through the AKT/mTOR signaling. Finally, decreased CYP2E1 expression was observed in the tumor tissues of HCC patients and its expression level in tumors was negatively correlated with the serum level of total bile acids (TBA) and gamma-glutamyltransferase (GGT).

**Conclusions:**

CYP2E1 downregulation contributes to CA-induced HCC development presumably through autophagy regulation. Thus, CYP2E1 may serve as a potential target for HCC drug development.

**Supplementary Information:**

The online version contains supplementary material available at 10.1186/s10020-024-00844-5.

## Introduction

Primary liver cancer is the fourth most diagnosed cancer and the second leading cause of cancer-related death in China with approximately 367,700 new cases and 316,500 deaths in 2022 (Han et al. 2024). Hepatocellular carcinoma (HCC) accounts for the majority (>80%) of liver cancers (Tamai et al. 2023). Risk factors for HCC include chronic infection with hepatitis B virus (HBV) or hepatitis C virus (HCV), aflatoxin-contained food, alcohol consumption, metabolic syndrome, etc. (Toh et al. 2023). Recently, bile acids (BAs) were also found to be involved in HCC development (Conde et al. 2021; Shen et al. 2022).

Bile acids are metabolites from cholesterol and synthesized mainly in the liver. BAs serve as endocrine signaling molecules sensed by nuclear or membrane-localized receptors to trigger specific signaling pathways and regulate many biological processes (Song et al. 2020; Cai et al. 2022). Studies from clinical patients and animal models have shown an association of BAs abnormalities with liver diseases such as HCC (Stepien et al. 2022). Clinically, increased levels of primary and secondary BAs were detected in HCC and cholangiocarcinoma carcinoma (CCA) patients (Changbumrung et al. 1990). Children with the progressive familial intrahepatic cholestasis type 2 (PFIC type 2) disease, characterized by a genetic deficiency of the canalicular bile salt export pump BSEP or ABCB11, and severe cholestasis with elevated serum and liver BAs levels, such as CA and chenodesoxycholic acid (CDCA), is predisposed to HCC (Knisely et al. 2006; Baptissart et al. 2013). The experiments in mice have shown that a CA-enriched diet strongly promoted N-nitrosodiethylamine-induced liver carcinogenesis (Yang et al. 2007). Our studies have also shown that CA administration promoted DEN-induced initiation and progression of HCC. However, the molecular mechanisms by which BAs promote HCC are still unknown.

Autophagy, a “self-eating” process that clears intracellular waste, is crucial for development, differentiation, survival, and homeostasis (Tabibzadeh 2023). Accumulating evidence indicates that autophagy can be upregulated in response to a multitude of stresses such as starvation, hypoxia, and intracellular pathogens, which in most contexts promotes tumorigenesis (Li et al. 2020). Increased autophagy activity with high LC3B expression benefits tumor proliferation, invasion, or metastasis (Wu et al. 2014). Autophagy is involved in HCC progression, resulting in drug resistance and poor prognosis of HCC patients (Shi et al. 2011; Li et al. 2020; Xu et al. 2020). The protective role of autophagy in cholestasis-induced liver injury was also reported (Gao et al. 2014). High levels of total bile acids (TBA) in HCC patients were found to be strongly correlated with their autophagy level and poor survival (Gao et al. 2019a). Autophagy triggered by glycochenodeoxycholate (GCDC) is a protective mechanism in the invasion and metastasis of HCC(Gao et al. 2019). How dysregulated levels of BAs influence autophagy, however, is still poorly understood.

Cytochrome P450 2E1 (CYP2E1), a member of the cytochrome P450 superfamily, is an important hepatic metabolic enzyme that is responsible for the metabolism of xenobiotics including ethanol, acetone, drugs, and procarcinogens (Torres et al. 2019; Yang et al. 2022). Research efforts have been focused on its role in drug metabolism and alcoholic liver diseases for a long time. However, CYP2E1 function in HCC development is largely unknown. Recent studies have shown that the expression level of CYP2E1 in the tumor tissues of HCC patients was significantly lower compared to the adjacent nontumor tissues and the cirrhotic and/or normal liver tissues (Ho et al. 2004). The level of CYP2E1 expression is negatively associated with aggressive tumor type and poor prognosis of HCC patients (Ho et al. 2004), suggesting the inhibitory activity of CYP2E1 in the initiation and progression of HCC. HepG2 cells overexpressing exogenous CYP2E1 exhibited a slow rate of cell growth accompanied with increased CYP2E1-induced cytotoxicity (Cederbaum et al. 2001; Alwadei et al. 2023). In the DEN-induced HCC rat model, CYP2E1 expression gradually declined along with the initiation, promotion, and progression of HCC (Sanchez-Meza et al. 2023). These studies indicate that downregulation of CYP2E1 expression is involved in HCC tumorigenesis.

In this study, we investigated CYP2E1 involvement in CA-induced autophagy and the promotion of malignant growth of hepatocellular carcinoma cells. We also examined CYP2E1 expression and TBA level in the tumor tissues of HCC patients.

## Materials and methods

### Chemicals and antibodies

CA was purchased from Sigma-Aldrich (C1129-500G, St Louis, MO, USA). MK-2206 dihydrochloride was obtained from MedChemExpress (HY-108,232, USA). Antibodies against p62 (18420-1-AP), CYP2E1 (19937-1-AP), AKT (10176-2-AP), and p-AKT (66444-1-lg) were from Proteintech Group (Chicago, IL, USA). The mTOR (380,411) antibody was from Zen BioScience (Chengdu, Sichuan, China). p-mTOR (#5536), LC3A/B (#12,741) and Alexa Fluor®594 Conjugate (#8889S) were from Cell Signaling Technology (Danvers, MA, USA). β-actin (AF0003) was purchased from Beyotime Biotechnology (Nanjing, Jiangsu, China). Alexa Fluor 488 (A21206) was from Sigma-Aldrich Co. (St. Louis, MO, USA). Chloroquine (CQ) was form MedChemExpress (HY-17,589 A, USA).

### Clinical tumor samples

HCC cancerous and para-cancerous tissues were obtained from Union Hospital, Tongji Medical College, Huazhong University of Science and Technology between May 2019 and December 2023. The samples were from forty men and six women aged 42–78 years (mean 61.7 years), and none of them had received any anticancer treatment before surgery.

### Cell culture and stable cell lines

Human HCC HepG2 and Huh7 cell lines were obtained from Shenogen Pharma Group (Beijing, China), and maintained in DMEM with 10% FBS at 37℃ in a humidified incubator with 5% CO_2_. Stable CYP2E1 expression cell lines and their control cell lines were generated by stable transfection of pcDNA-CYP2E1 and pcDNA empty vector (Fenghui Biotechnology Co., LTD) respectively. Then, CYP2E1 expression in the stable cell lines were verified by Western blot analysis.

### Western blot analysis

Cells and liver tissues were lysed with the RIPA buffer. Proteins were separated through SDS-PAGE, transferred onto a PVDF membrane that was blocked and incubated with the indicated antibody. Target proteins were detected by the ECL assay System.

### Animal experiments

Male BALB/c-nu mice (5 weeks old) were purchased from Beijing SiPeiFu Animal Technology Co. LTD and housed in specific pathogen-free conditions with the constant temperature (24 ± 2℃) and relative humidity (60%). HepG2-CYP2E1 cells and HepG2-vector cells (5 × 10^6^ cells/mice) were injected into the liver of BALB/c-nu mice (*n* = 4) to establish orthotopic liver xenograft tumor model with or without intragastric administration of CA (0.2%, every 2 days) and maintained for 28 days. Tumor volume was calculated according to the following formula: tumor volume = (major axis) × (minor axis)^2^/2. Male Sprague-Dawley (SD) rats, 6 weeks old (~ 230–250 g), were obtained from the Hunan SJA Laboratory Animal Co., LTD, China. Rats were intraperitoneally injected with DEN (Sigma Chemical Co., LTD) of 75 mg/kg per body weight once a week for three weeks, then with DEN of 100 mg/kg per body weight once a week for three weeks (Matsuzaki et al. 1992; Shiota et al. 1999). All rats were subsequently fed with or without CA for 23 weeks. After euthanization, the livers were removed. Preparation and measurement of liver tumors were performed by Biotree Biotech Co., LTD., Shanghai, China as a custom service.

### Immunofluorescence staining

After fixing with ice-cold 4% paraformaldehyde for 15 min, and permeabilizing with 0.1% Triton X-100 for 20 min, the samples were incubated with LC3 or p62 antibodies overnight at 4 ℃, washed and then incubated with the Alexa Fluor conjugated secondary antibody for 1 h. The nuclei were stained with Hoechst 33,342 (1 µg/mL) for 10 min. The images were obtained using a fluorescence microscope (Leica TCS SP8).

### pmCherry-EGFP-LC3 puncta assay

Cells were seeded in a 24-well plate, infected with the pmCherryEGFP-LC3b adenovirus (MiaoLingbio, China) for 6 h, incubated with CA for 24 h and examined with a confocal microscope (Leica TCS SP8).

### Colony formation assay

After seeding (4 × 10^3^ cells/well), cells were treated with CA and cultured for 2 weeks. Then, the cell colonies were washed, fixed and stained with 0.1% crystal violet. The number of colonies were counted under a microscope.

### Immunohistochemistry staining

After deparaffinized, rehydrating gradually by gradient alcohol and incubating in a 3% hydrogen peroxide/methanol buffer, the slides were immersed in an ethylenediamine tetraacetic acid buffer (pH 8.0) and boiled for 5 min. Then, the slides were washed and incubated with the specific primary and secondary antibodies. Diaminobenzidine was used to generate the signal, and images were captured.

### Gene and protein expression analysis

GEPIA and UALCAN programs (http://ualcan.path.uab.edu) were used to analyze the relative mRNA expression of CYP2E1 in the tumor and normal samples of HCC patients. The protein expression was analyzed using the data from the Clinical Proteomic Tumor Analysis Consortium (CPTAC, http://ualcan.path.uab.edu/analysis-prot.html). The GEPIA2.0 and Kaplan-Meier programs were then used for patient survival analysis.

### Statistical analysis

Data were presented as the mean ± standard error of the mean (SEM) using a GraphPad Prim 8.0 software with analysis of variance (ANOVA), followed by Bonferroni’s post hoc tests. Statistical significance was set at *p* < 0.05. Spearman correlation coefficient was used to determine the correlations between abnormal distribution variables.

## Results

### CYP2E1 expression is downregulated in CA-treated hepatocellular carcinoma induced by DEN

**Fig. 1:**
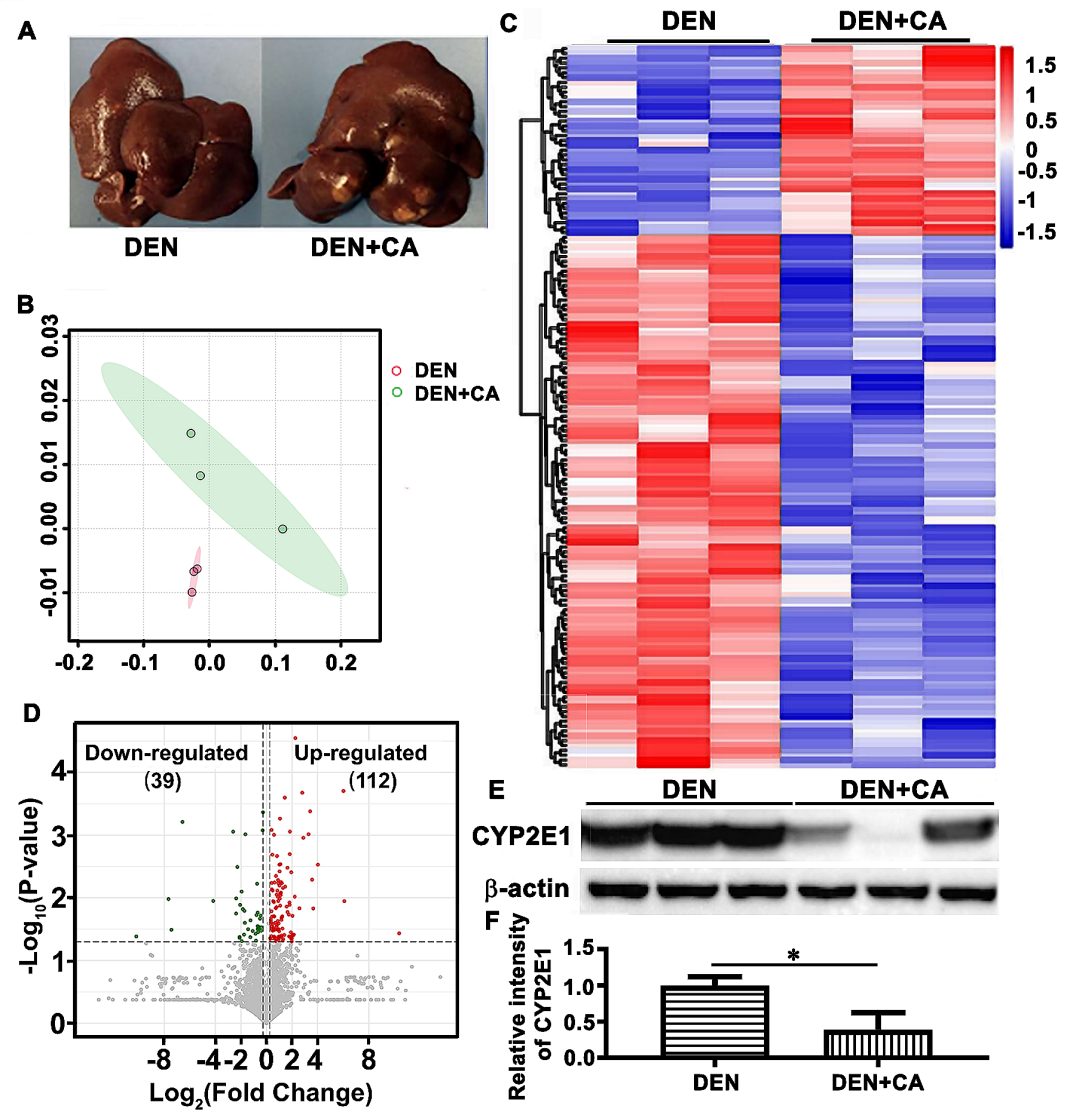
CA treatment decrease CYP2E1 level in HCC tumors induced by DEN. (**A**) Representative images of the livers from the male SD rats treated with DEN and then administrated with CA (group DEN + CA) and vehicle DMSO as a control (group DEN). (**B-D**) The general proteome information. (**B**) Principal Coordinates Analysis (PCoA) showed a clear separation of proteins between the group DEN and the group DEN + CA. (**C**) 151 differential proteins were identified in the tumor tissues of the group DEN + CA vs. the group DEN, (*p < 0.05, increased proteins: red; decreased proteins: blue). (**D**) The increased (red) or decreased (blue) expression of proteins in the group DEN + CA vs. the group DEN (*p < 0.05) were shown. (**E-F**) CYP2E1 protein expression from individual rats (*n* = 3) were validated by Western blot analysis (**E**) and then quantitatively analyzed (**F**). The data represent the mean ± SEM. **p < 0.01

Our recent studies found that CA administration promoted DEN-induced initiation and progression of HCC in rats. To explore the molecular mechanisms by which CA promotes hepatocellular carcinogenesis, liver tumor tissues from the DEN-treated, and DEN together with CA treated rats were used for whole-proteome label-free assessment (Fig. 1A). Principal coordinates analysis (PCoA) showed a clear separation of proteins between these two groups (Fig. 1B). 151 differential proteins with 112 proteins upregulated and 39 proteins downregulated were detected between the tumor tissues from group DEN + CA and group DEN (Fig. 1C-D). The differentially expressed proteins between these two groups were selected according to the criteria of (fold-change ≥ 1.2 or ≤ 0.83, respectively, and *p* < 0.05). Volcano plot and heatmap were shown in Fig. 1C and D. Further analysis with the Protein-Protein Interaction Network Analysis using the STRING database indicated involvement of CYP2E1 in three out of five enriched KEGG pathways (Fig S1A). CYP2E1 expression was significantly downregulated in the group DEN + CA rats (> 2-fold) compared to the DEN group. In Western blot analysis, we found that CYP2E1 expression was significantly downregulated in the liver tumors from the group DEN + CA compared to those in the group DEN (Fig. 1E-F), consistent with the proteomics data.

**Fig. 2:**
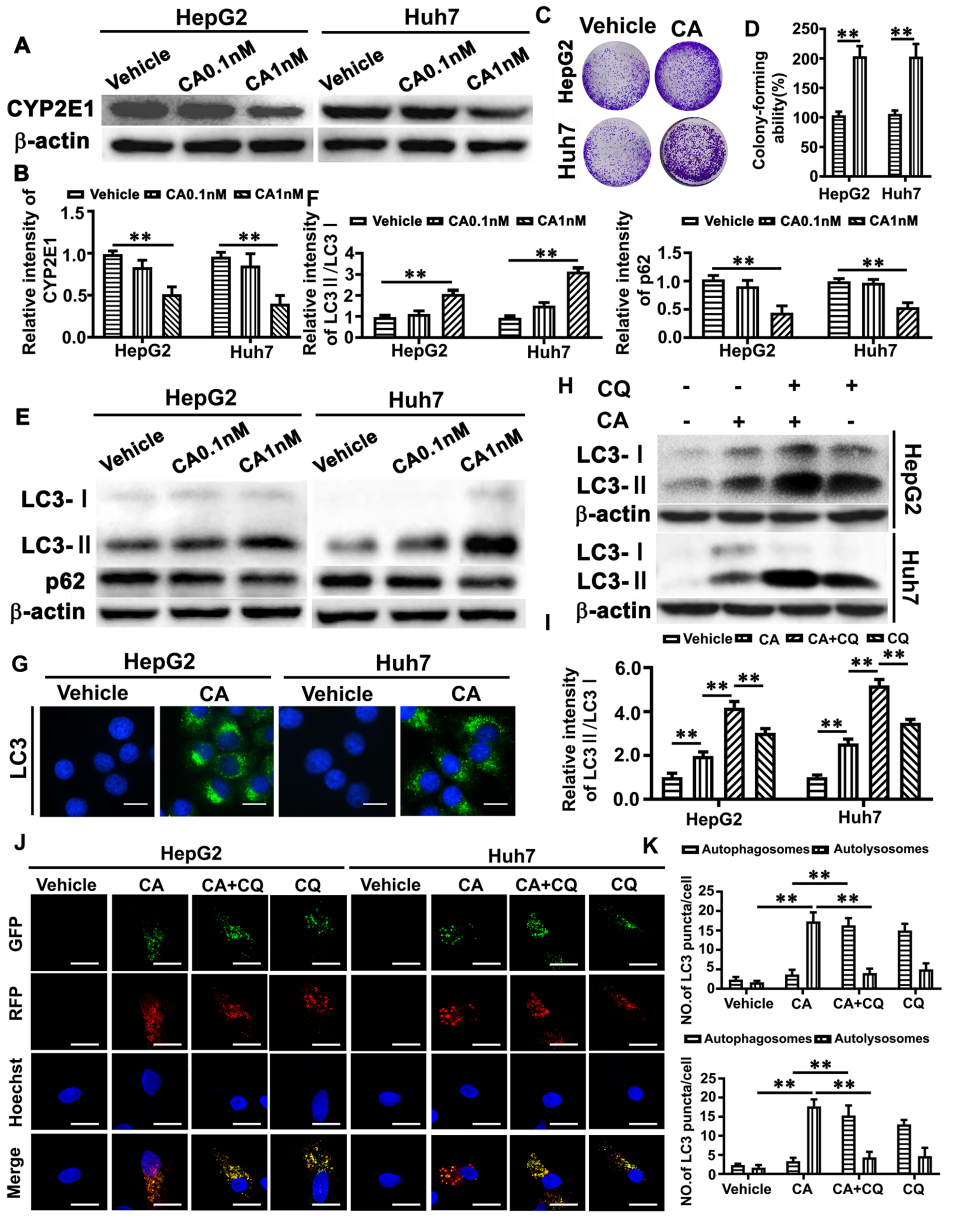
CA treatment decreases CYP2E1 expression and activates autophagy in both HepG2 and Huh7 cells. (**A-B**) HCC cells were treated with 0.1 and 1nM CA for 24 h and the same volume of DMSO was used as a vehicle control. CYP2E1 expression was assessed by Western blot analysis (**A**), and then quantitatively analyzed (**B**). The data represent the mean ± SEM (*n* = 3). (**C-D**) HCC cells were treated with CA (1 nM) for 14 days, and the representative images of colony formation were shown (**C**) and then quantitatively analyzed (**D**). (**E-F**) HCC cells were treated with 0.1 and 1nM CA for 24 h and the same volume of DMSO was used as a vehicle control. The expression of LC3II/LC3I and p62 were assessed by Western blot analysis (**E**) and then quantitatively analyzed (**F**). The data represent the mean ± SEM. **p < 0.01. (**G**) HCC cells were treated with CA (1nM) for 24 h and analyzed with immunofluorescence assay using the LC3II antibody (488 green). Hoechst (blue) was used to stain the nuclei, and the stained HCC cells were then photographed under a fluorescence microscope. The scale bar is 20 μm. (**H-I**) HCC cells were treated with CA (1nM), supplemented with or without Chloroquine (CQ, 20µM) for 24 h. The expression level of LC3II/LC3I was assessed by Western blot analysis (**H**) and then quantitatively analyzed (**I**). (**J**) The HCC cells were infected with the pmCherry-EGFP-LC3b adenovirus, and then treated with CA (1nM), supplemented with or without CQ (20µM) for 24 h, and analyzed by confocal microscopy. The scale bar is 10 μm. (**K**) The quantification of autophagosomes (yellow puncta) and autolysosomes (red puncta) was shown. **p < 0.01

To examine whether CA regulates CYP2E1 expression, HepG2 and Huh7 cells were treated with different concentrations of CA for 24 h. Western blot analysis revealed that CA treatment reduced CYP2E1 expression in a dose-dependent manner (Fig. 2A-B). In colony formation assays, CA significantly increased the colony formation of HCC cells at a concentration of 1 nM, compared to the vehicle control (Fig. 2C-D). These results suggested that CA at the concentration of 1 nM was able to downregulate CYP2E1 expression and promote the growth of HepG2 and Huh7 cells.

### CYP2E1 downregulation contributes to CA-induced HCC growth through autophagy regulation

Bile acids such as glycochenodeoxycholate (GCDC) have been reported to induce autophagy, which promotes the invasion and migration of HCC cells (Gao et al. 2019). To determine the role of CA in autophagic regulation, the levels of LC3II/I (a marker of autophagosome) and p62 (a marker of autophagic flux) expression were examined in HepG2 and Huh7 cells treated with different concentrations of CA for 24 h. In Western blot analysis, we found that CA treatment upregulated the LC3II/I expression while downregulated p62 expression (Fig. 2E-F). We also found that the HCC cells treated with CA at the concentration of 1 nM exhibited more LC3 puncta, compared to vehicle control treated cells (Fig. 2G). The increased level of LC3II/I and number of LC3 puncta can be related to either increased formation of autophagosomes or impaired degradation of autophagosomes. We further evaluated the autophagic flux. When an autophagosome-lysosome fusion inhibitor (late autophagy inhibitor, CQ) was used, we found that CQ enhanced the CA-induced LC3II/I expression in HCC cells (Fig. 2H-I). In addition, we infected HepG2 and Huh7 cells with the pmCherry-EGFP-LC3b adenovirus to monitor the synthesis of autophagosomes (yellow puncta) and autophagosome-lysosomal fusion (red puncta). Many red puncta with a few yellow puncta were observed in CA-treated HCC cells. In contrast, CQ treatment markedly increased the number of yellow puncta in HCC cells (Fig. 2J-K). Co-treatment of HCC cells with CA and CQ led to an increase in yellow puncta compared with CA treatment alone (Fig. 2J-K). These results thus indicate that CA induces autophagy.

**Fig. 3:**
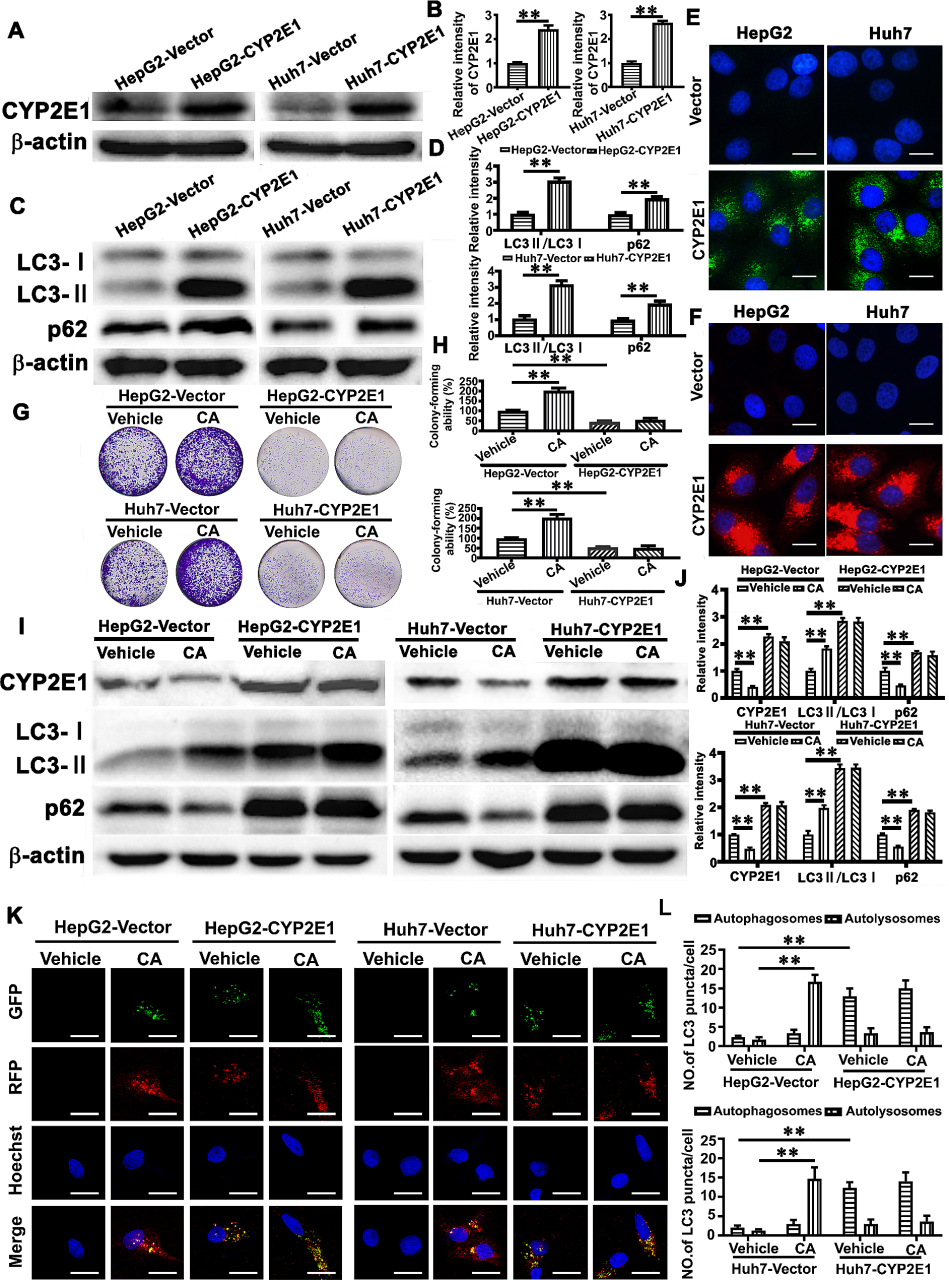
CYP2E1 deficit contributes to CA-induced HCC growth with mechanism involving regulating autophagy. (**A-B**) The level of CYP2E1 expression in the HepG2 and Huh7 cells with forced expression of CYP2E1 or vector control was measured by Western blot analysis (**A**) and quantitatively analyzed (**B**). (**C-D**) The levels of LC3II/LC3I and p62 in the HCC cells with forced expression of CYP2E1 or vector control were measured with Western blot analysis (**C**) and quantitatively analyzed (**D**). (**E-F**) HCC cells with forced expression of CYP2E1 or vector control were analyzed with immunofluorescence assay using the LC3II antibody (488 green) (**E**) and p62 antibody (594 red) (**F**). Hoechst (blue) was used to stain the nuclei, and the stained HCC cells were then photographed under a fluorescence microscope. The scale bar is 20 μm. The HCC cells with forced expression of CYP2E1 or vector control were treated with or without CA (1 nM) for 14 days, and the representative images of colony formation were shown (**G**) and then quantitatively analyzed (**H**). (**I-J**) HCC cells with forced expression of CYP2E1 or vector control were treated with or without CA (1nM) for 24 h and the same volume of DMSO was used as a vehicle control. The levels of CYP2E1, LC3II/LC3I and p62 expression were assessed by Western blot analysis (**I**) and then quantitatively analyzed (**J**). The expression levels of CYP2E1 and p62 were normalized against that of β-actin. The data represent the mean ± SEM. **p < 0.01. (**K**) The HCC cells with forced expression of CYP2E1 or vector control were infected with the pmCherry-EGFP-LC3b adenovirus, and then treated with or without CA (1nM) for 24 h, and analyzed by confocal microscopy. The scale bar is 10 μm. (**L**) The quantification of autophagosomes (yellow puncta) and autolysosomes (red puncta) was shown. **p < 0.01

To further confirm the role of CYP2E1 in CA-induced autophagy and growth of HCC cells, we established the HepG2 and Huh7 cells with forced expression of CYP2E1(Fig. 3A-B). The colony formation assay showed that high level of CYP2E1 expression attenuated the growth of HCC cells (Fig. 3G-H). Western blot analysis revealed the cells with forced expression of CYP2E1 exhibited increased expression level of LC3II/I (Fig. 3C-D), and immunofluorescence staining also showed increased LC3 puncta (Fig. 3E), suggesting that forced expression of CYP2E1 induces the accumulation of autophagosome in HCC cells. In addition, the cells with forced expression of CYP2E1 increased the expression level of p62 as shown by Western blot analysis (Fig. 3C-D) and augmented the accumulation of p62 points by immunofluorescence staining (Fig. 3F). Moreover, HepG2 and Huh7 cells were infected with the pmCherry-EGFP-LC3b adenovirus, and increased yellow puncta (autophagosomes) were detected in HepG2 and Huh7 cells with forced expression of CYP2E1 (Fig. 3K-L). These results thus indicated that increased level of CYP2E1 in HCC cells attenuated cell growth and blocked autophagic flux which thus resulted in autophagosome accumulation. Next, HCC cells with or without forced CYP2E1 expression were treated with CA for 24 h, and we found that CA failed to increase the accumulation of autophagosome, autophagic flux and cell growth in the HCC cells with forced CYP2E1 expression, which were obviously observed in the HCC cells without forced CYP2E1 expression (Fig. 3G-L), indicating that CYP2E1 plays an important role in CA-induced growth of HCC cells and regulation of autophagy.

### AKT/mTOR signaling pathway is involved in CA-induced autophagy and cell growth of HCC regulated by CYP2E1

**Fig. 4:**
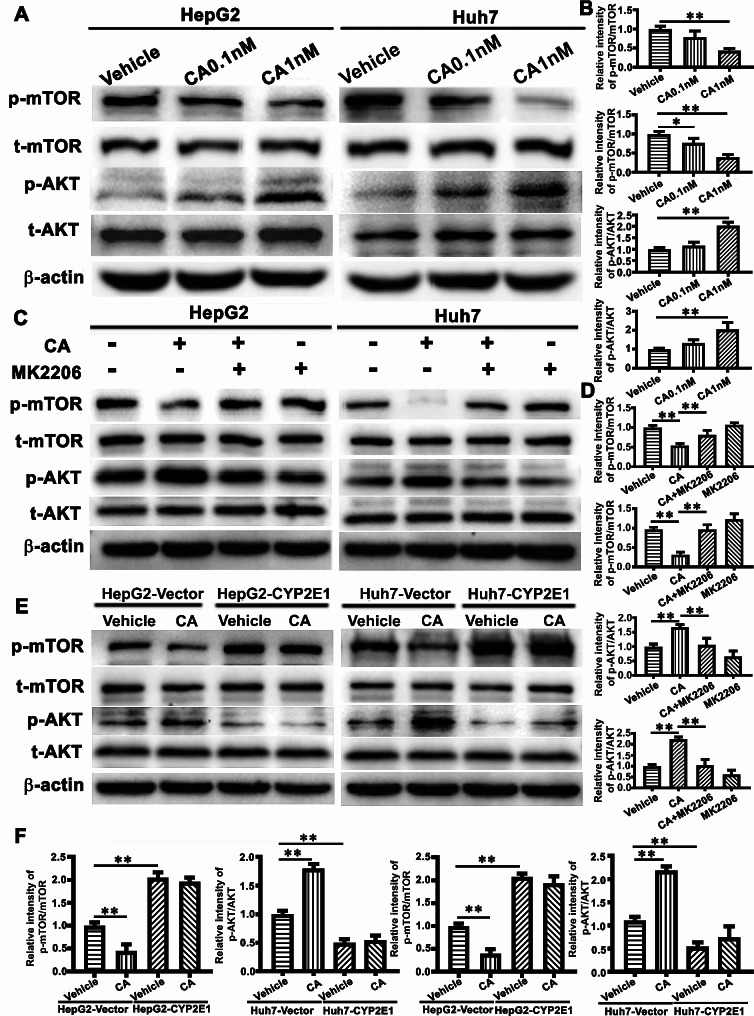
The AKT/mTOR signaling pathway is involved in CA-induced autophagy and growth of HCC cells regulated by CYP2E1. (**A-B**) HCC cells were treated with 0.1 and 1nM cell CA for 24 h and the same volume of DMSO was used as a vehicle control. The levels of p-mTOR, mTOR, p-AKT and AKT were detected by Western blot analysis (**A**) and quantitatively analyzed (**B**). The levels of phosphorylated AKT and mTOR were normalized against the AKT and mTOR, respectively. (**C-D**) The HCC cells were exposed to MK2206 (20 µM) for 2 h, followed by CA (1nM) treatment for 24 h, and subsequently the expression levels of p-mTOR, mTOR, p-AKT and AKT were assessed by Western blot analysis (**C**) and then quantitatively analyzed (**D**). (**E-F**). The HCC cells with forced expression of CYP2E1 or vector were treated with or without CA (1nM) for 24 h and the same volume of DMSO was used as a vehicle control. The levels of p-mTOR, mTOR, p-AKT and AKT were assessed by Western blot analysis (**E**) and then quantitatively analyzed (**F**). The data represent the mean ± SEM. **p < 0.01

As the AKT/mTOR signaling plays a central role in the regulation of tumor growth and autophagy, we measured the phosphorylated levels of AKT and mTOR in the CA treatment of HCC cells using Western blot analysis with phosphorylation-specific antibodies. Figure 4A-B showed that CA upregulated the level of AKT phosphorylation, while downregulated the level of the mTOR phosphorylation. In addition, MK2206, an AKT inhibitor, significantly increased p62 expression (Fig S2A-B) and mTOR phosphorylation in the CA-treated cells, and inhibited the colony-forming ability of the HCC cells induced by CA treatment (Fig S2C-D), suggesting that AKT/mTOR pathway is involved in CA-induced autophagy and promotion of HCC cell growth (Fig. 4C-D).

Since AKT phosphorylation was previously reported to be suppressed by CYP2E1 in chronic ethanol-induced fatty liver(Zeng et al. 2018). To determine whether CYP2E1 is involved in CA-activation of the AKT/mTOR signaling pathway, HCC cells with or without forced expression of CYP2E1 were treated with CA, and phosphorylation of AKT and mTOR was measured. We found that forced expression of CYP2E1 downregulated the AKT phosphorylation whereas upregulated mTOR phosphorylation, regardless CA treatment (Fig. 4E-F). Consistently, as shown in Fig. 3G-L, CA failed to influence the accumulation of autophagosome, autophagic flux and cell growth in the HCC cells with forced CYP2E1 expression. Taken together, these results indicate that CYP2E1 plays an important role in CA-induced growth of HCC cells presumably through regulation of autophagy and the AKT/mTOR signaling pathway.

### CYP2E1 regulation of the AKT/mTOR pathway is involved in CA-promoted growth of HCC cells in vivo

**Fig. 5:**
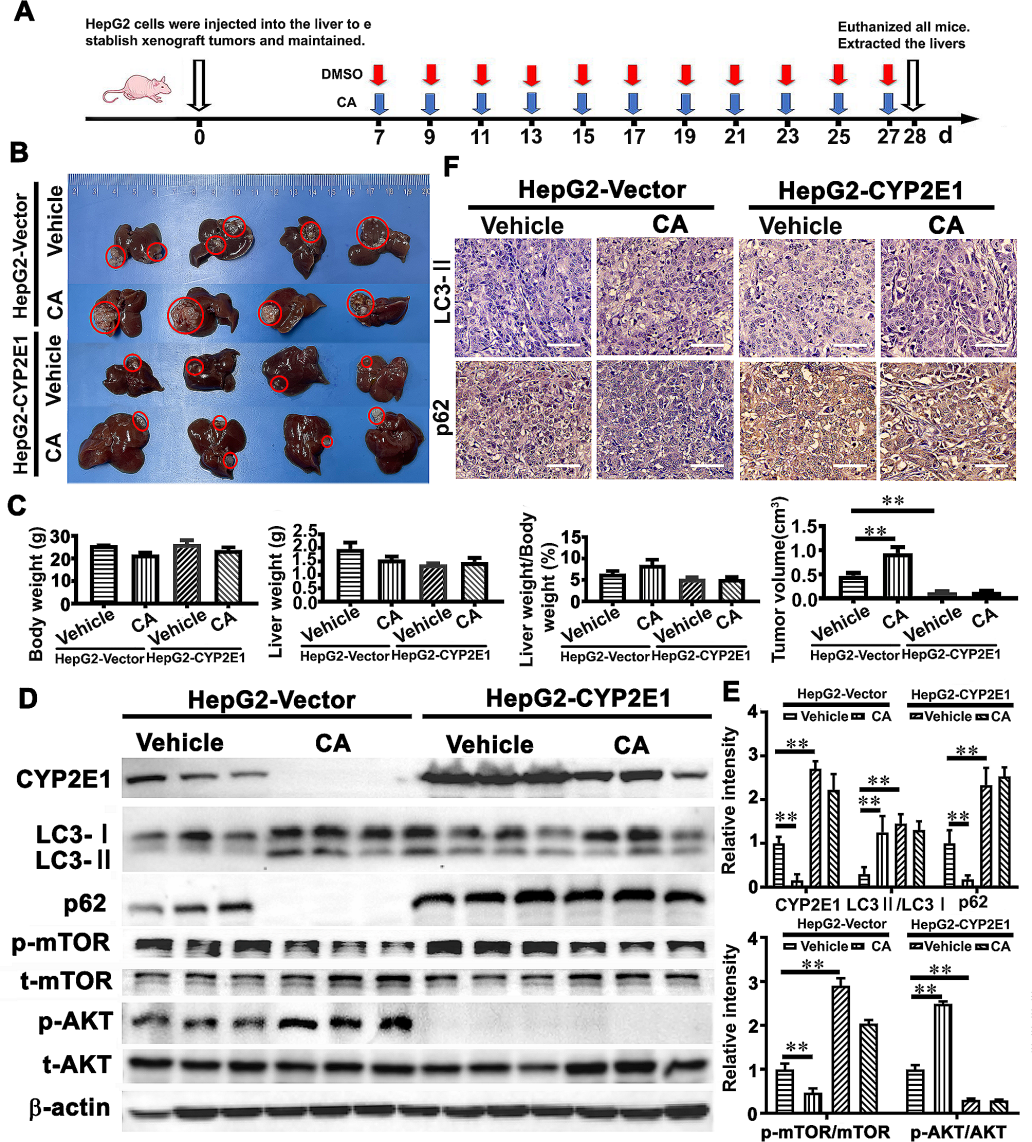
CYP2E1 is involved in CA-induced promotion of HCC cell growth in nude mice. The nude mice were intrahepaticly injected with HepG2 cells with forced expression of CYP2E1 or vector control, and then intragastrically administrated with CA or the same volume of DMSO as a control for 28 days (**A**). Representative image of liver tumors in these nude mice (**B**) and quantitative analysis of liver tumor volumes, liver weight, body weight and the ratio of liver weight to body weight of the nude mice (**C**) were shown. (**D-E**) Expression levels of CYP2E1, LC3II/LC3I and p62 and the levels of the phosphorylated AKT and mTOR in the liver tumors from the nude mice with or without CA administration were assessed by Western blot analysis (**D**) and then quantitatively analyzed (**E**). β-actin was used as the internal loading control. The levels of phosphorylated AKT and mTOR were normalized against the AKT and mTOR, respectively. The data represent the mean ± SEM. **p < 0.01. The expression levels LC3II and p62 in the liver tumors from the nude mice with or without CA administration were examined by immunohistochemical staining (**F**). The scale bar is 50 μm

To further assess the role of CYP2E1 in CA-induced hepatocellular carcinogenesis, we performed a xenograft experiment in nude mice. The nude mice were intrahepatically injected with the HepG2 cells with forced CYP2E1 expression (HepG2-CYP2E1) and a control cell line of the HepG2 cells transfected with an empty expression vector (HepG2-Vector), and maintained with or without intragastric supplement of CA for 28 days (Fig. 5A). We found that tumors were formed in the livers of all mice, and the tumor volumes formed by the HepG2-Vector cells in the groups supplemented with CA were bigger compared with the group with the same volume of vehicle. The tumor volumes formed by the HepG2-CYP2E1 cells were much smaller than that of the HepG2-Vector cells. However, there were no changes between the HepG2-CYP2E1 groups with or without CA administration (Fig. 5B-C), suggesting that the upregulation of CYP2E1 is a critical mechanism by which CA influences HCC growth. Liver weights, body weight and the ratio of liver weight to body weight were not changed in all groups (Fig. 5B-C). Western blot analysis revealed that CA increased the levels of LC3II/I expression and AKT phosphorylation whereas decreased the levels of p62 expression and mTOR phosphorylation in the tumors formed by the HepG2-Vector cells (Fig. 5D-E). Compared to the tumors formed by the HepG2-Vector cells, tumor tissues formed by the HepG2-CYP2E1 cells showed increased levels of LC3II/I and p62 expression and mTOR phosphorylation, and decreased levels of AKT phosphorylation, but the expression and phosphorylation of these proteins were without any changes after CA administration (Fig. 5D-E). Similar results were observed by IHC staining (Fig. 5F). These results thus indicated that CA administration accelerated tumor growth of HepG2 cells through the AKT/mTOR pathway regulated by CYP2E1.

### Low level of CYP2E1 expression correlates with poor progress and increased TBA as well as GGT in HCC patients

**Fig. 6:**
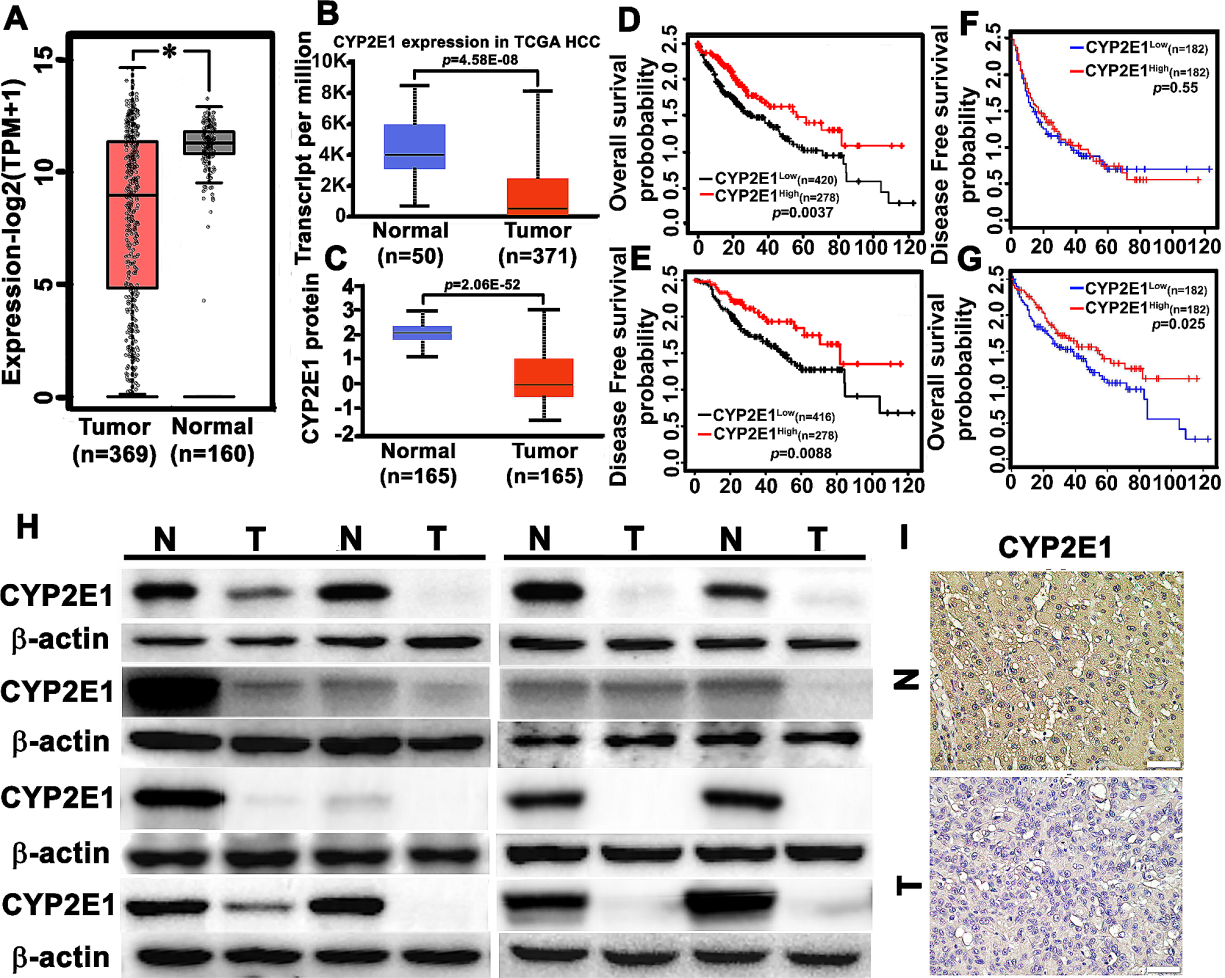
Low expression of CYP2E1 correlates with poor progress and increased levels of TBA and GGT in HCC patients. (**A**) Comparison of the CYP2E1 mRNA level across TCGA tumors, and the matched TCGA normal and GTEx data were included as controls. The box plot data were supplied. *p < 0.05. (**B**) Comparison of the CYP2E1 mRNA level between HCC and normal tissues in TCGA database. Statistical significance was assessed using two-tailed Student’s t-test. (**C**) CYP2E1 proteomic expression profile in HCC from CPTAC samples. Z-values represent standard deviations from the median across samples for HCC. N represents the number of samples. (**D**, **E**) Kaplan-Meier analysis of overall survival (**D**) and disease-free survival probability (**E**) of CYP2E1 levels in HCC patients. The statistical significance was assessed using two-sided log-rank test according to HCC patients with low or high expression of CYP2E1. (**F**, **G**) The overall survival (**F**) and disease-free survival probability (**G**) were compared between CYP2E1 high and low expression in the HCC patients from TCGA cohort. (**H**, **I**) The level of CYP2E1 expression in the specimens of HCC tumor tissues (**T**) and adjacent non-tumor tissues (**N**) was assessed with Western blot analysis (**H**) and immunohistochemical staining (**I**)

To reveal the clinical relevance of CYP2E1 expression with clinical features of HCC patients, TCGA (The Cancer Genome Atlas) and GEPIA (Gene Expression Profiling Interactive Analysis) were used to evaluate CYP2E1 expression between the normal and tumor tissues of HCC patients. We found that CYP2E1 mRNA expression was lower in the tumor tissues than in the normal tissues (Fig. 6A-B). The difference of the CYP2E1 protein between the tumor and normal tissues in HCC was also analyzed using the data from the Clinical Proteomic Tumor Analysis Consortium (CPTAC). We found decreased CYP2E1 protein expression in the tumor tissues compared with the normal tissues in HCC patients (Fig. 6C). Then, we assessed the differences in the survival risk between the high CYP2E1 expression and low CYP2E1 expression using the Kaplan-Meier (Fig. 6D-E) and GEPIA (Fig. 6F-G) survival analysis and found that the HCC patients with low CYP2E1 expression exhibited worse disease-free survival and overall survival rates. Thus, our results suggested that downregulation of CYP2E1 contributes to HCC development, and may serve as an important indicator for poor prognosis in HCC patients.

To confirm this, we examined CYP2E1 expression in forty-six cases of HCC specimens with Western blot analysis and IHC. Compared with the paired normal tissues, lower CYP2E1 expression was detected in 40 of 46 (86.96%) tumor specimens compared to the neighboring normal tissues (Fig. 6H-I; Table 1). In addition, CYP2E1 expression was correlated with the serum levels of TBA and GGT (*p* < 0.05; Table 1), but not with AST, ALT, ALP or AFP (*p* > 0.05). Taken together, these findings indicate that downregulated CYP2E1 expression and increased serum TBA as well as GGT are involved in hepatocellular carcinogenesis.

**Table 1:** Correlations between CYP2E1 level and liver function test in HCC patients

Factors	CYP2E1		*p*-value
	Low	High	
**TBA**			0.009**
> 10	23	0	
≤ 10	17	6	
**ALT**			0.171
> 40	10	0	
5 ~ 40	30	6	
**AST**			0.314
> 40	6	0	
8 ~ 40	34	6	
**GGT**			0.031*
> 50	23	1	
11 ~ 50	17	5	
**ALP**			0.628
> 150	4	1	
≤ 150	36	5	
**AFP**			0.462
> 20	18	2	
≤ 20	22	4	
**TBIL**			0.756
> 19	9	2	
5 ~ 19	31	4	

*p < 0.05, and **p < 0.01 were considered statistically significant

## Discussion

Our study demonstrated that CA treatment decreased CYP2E1 expression and promoted the malignant growth of human HCC HepG2 and Huh7 cells both in vitro and in vivo. The tumor promotion activity of CA in these cells was associated with CYP2E1-regulated induction of autophagy. We also found that high levels of TBA in HCC patients were strongly correlated with decreased CYP2E1 level. Hence, the CYP2E1-regulated autophagy is one of the underlying mechanisms of CA action in HCC.

Studies have shown that BAs homeostasis was disturbed during HCC development. A retrospective cohort study identified persistently elevated TBA as a major independent risk factor for HCC development in chronic hepatitis B patients (Wang et al. 2016). Previously, bile acids were considered as tumor promoters and involved in the pathogenesis of HCC (Ma et al. 2018; Shen et al. 2022). Patients with cholestasis, a chronic and consistent exposure to BAs, often develop liver fibrosis and cirrhosis which eventually results in liver failure and increased risk of HCC or CCA (Eaton et al. 2013; Tomiyama et al. 2013). The increased levels of BAs, such as CA and CDCA in cholestatic liver diseases induce oxidative stress and apoptosis, thereby resulting in damage to the liver parenchyma and, eventually extrahepatic tissues (Monte et al. 2009). The unconjugated BAs including CA, CDCA, and DCA, are cytotoxic, and their accumulation could result in mitochondrial damage, disruption of cell membranes, production of reactive oxygen species (ROS) and induction of DNA damage and mutation in hepatocytes (Jang et al. 2012; Guicciardi et al. 2013). Toxic BAs-induced chronic inflammation and injury-repair response in the liver that likely contributes to tumor promotion (Li and Apte 2015). In this study, we also found that high levels of TBA were detected in 50% (23/46) cases of HCC patients, and increased level of TBA was correlated with decreased level of CYP2E1 expression. CYP2E1 is a key metabolic enzyme of the liver microsomal oxidase system and is involved in the induction of oxidative stress(Cho et al. 2021). We chose CA for this study mainly because it is one of the major primary BAs produced in the liver and was increased in our DEN-induced HCC model. We used liver tumor tissues from DEN-induced liver tumor models with or without CA administration for whole-proteome label-free assessment and found that CA administration increased the tumor volumes and decreased the level of CYP2E1 expression. HepG2 and Huh7 cells treated with CA and nude mice intrahepatically injected with human HCC HepG2 cells with or without CA administration for 28 days exhibited increased cell growth and decreased CYP2E1 expression. Thus, a possible relationship between CA and decreased CYP2E1 expression was suggested.

Cytochrome P450 2E1 (CYP2E1) is mainly expressed in the liver and plays a critical role in the metabolism of many environmental toxicants as well as cancer inducing agents, such as benzene, ethanol, carbon tetrachloride, and vinyl chloride (Gonzalez 2005; Kang et al. 2007; Hu et al. 2023; Yan et al. 2023). CYP2E1 is involved in enhanced activation of procarcinogens to carcinogens (Harjumaki et al. 2021) as well as induction of oxidative stress (Cho et al. 2021), and it is suggested to be associated with the risk of liver cancer (Shun-Zhang Yu 2002). CYP2E1 expression is downregulated in tumor tissue of HCC patients, which is often associated with aggressive tumor type and poor prognosis of the patients (Zhu et al. 2022). By IHC, the mean CYP2E1 score was significantly lower in the livers with cirrhosis and HCC compared to normal livers, and it was considered that CYP2E1 might be directly induced by the factor(s) derived from component cells of tumors such as carcinoma cells themselves, whether primary or metastatic, stroma, and inflammatory cells (Hata et al. 2010). The results from the DEN-induced HCC model also showed that, CYP2E1 expression in the liver tissues was slightly higher than that in normal rat liver in the first to third week, which may be explained by the fact that CYP2E1 is required in the metabolism of nitrosamines. With the aggravation of liver lesions and development of cirrhosis, CYP2E1 expression was gradually downregulated. Here, we found that the level of CYP2E1 expression was decreased in forty out of forty-six tumor specimens compared to the adjacent nontumor tissues as revealed by Western blot analysis, suggesting that the decreased expression of CYP2E1 may be involved in HCC tumorigenesis. A decrease in carcinogen metabolism (Liu et al. 2022) and an increase in procarcinogen activation have also been documented as HCC risk factors as well as changes in the metabolism of environmental toxins that arise from alterations in Cytochrome P450 (CYP) activity (Forrester et al. 1990; Guengerich 1992; Guengerich et al. 1996; Fontham et al. 2009; Cheng et al. 2022). CYPs function not only in the detoxification of internal and external xenobiotics, but also in the metabolic activation of carcinogens, which may be further implicated in tumor initiation, promotion, and progression (Singh et al. 2023). Previously, the functional characterization of CYP2E1 has been focused on its role in alcoholic liver diseases and drug metabolism (Nagappan et al. 2019), since it metabolizes and activates many toxicologically important compounds, such as aromatic hydrocarbons such as ethanol, carbon tetrachloride, acetaminophen, benzene, halothane, and many other halogenated substrates (Chen et al. 2019; Cui et al. 2019; Cho et al. 2023). However, the function of CYP2E1 in hepatocarcinogenesis is poorly known. Our proteomic analysis of liver tumor tissues from DEN-induced male SD rats with CA administration revealed that CA treatment downregulated CYP2E1. Thus CYP2E1 was selected for further investigation because of its expression level (fold changed ≥ 2) and degree (the number of connections ≥ 3) revealed by Protein-Protein Interaction Network Analysis, and its involvement in three out of the top five enriched KEGG pathways. CYP2E1 is an important hepatic metabolic enzyme that might play an important role in the pathogenesis of CA-induced hepatocellular carcinogenesis. Here, we found that CYP2E1 is involved in CA-induced promotion of malignant growth in HCC cells by activation of autophagy in vitro and in vivo. Forced CYP2E1 expression inhibited cell growth of HCC accompanied by autophagy inhibition. However, in the HCC cells with forced expression of CYP2E1, CA failed to influence CYP2E1 level and autophagy.

Dysregulation of autophagy is involved in numerous diseases such as neurodegenerative disease, metabolic diseases, and cancer (Beckers, Tharkeshwar, and Van Damme 2021; Luo et al. 2021). Tumor cells upregulate autophagy to support their elevated metabolic demand for proliferation, survival, and malignancy (Amaravadi et al. 2016; Kimmelman and White 2017; Poillet-Perez and White 2019). Several signaling pathways are known to regulate autophagy including the mTOR, the PI3K-AKT, and the MAPK/ERK1/2 signaling pathways, and mTOR is one of the main negative regulators of autophagy (Wang et al. 2017; Gao et al. 2019; Zhang et al. 2019). In this study, we found that autophagy is activated by CA, which is involved in CA’s role in the promotion of HCC cell growth. CA upregulated the AKT phosphorylation but downregulated the mTOR phosphorylation. Previously, it was reported that AKT phosphorylation was induced by CYP2E1-mediated oxidative stress (Zeng et al. 2018). The interplay between ROS and autophagy in tumor cell functions from tumor initiation to progression has been widely reported(Xing et al. 2022). During the initiation, progression and metastasis of tumor cells, the pro-tumoral role of autophagy was shown to eliminate ROS-induced metabolic stress and the production of nutrients required for tumor cell survival (Gao et al. 2019). CYP2E1, a key enzyme for the generation of ROS, is a major contributor to the pathogenesis of many liver diseases such as alcoholic liver disease and HCC (Gao et al. 2019), but reports of its role in autophagy regulation are limited. Here, we found that forced CYP2E1 expression increased the accumulation of autophagosomes as revealed by increased LC3 puncta, and blocked autophagic flux with increased p62 accumulation and blockage of autophagosome-lysosome fusion both in vitro and in vivo. Recent studies have shown that inhibition of autophagic flux resulted in the accumulation of damaged organelles and dysfunctional proteins, which has the potential to turn autophagy into a serious and destructive process, leading to fatal toxic effects on tumor cells, providing an explanation of the inhibitory activity of CYP2E1 in HCC cell growth. We also found that CYP2E1-induced autophagy and regulation of the AKT/mTOR signaling are involved in CA-promoted cell growth of HCC.

## Conclusions

Our study revealed that CYP2E1 regulates CA-induced autophagy and promotion of HCC cell growth by regulating the AKT/mTOR signaling both in vitro and in vivo. Clinically, high TBA level in HCC tissue was strongly associated with decreased expression of CYP2E1. Targeting CYP2E1-mediated autophagy may be a novel and attractive therapeutic approach for amelioration of HCC with imbalance of BAs level.

### Electronic supplementary material

Below is the link to the electronic supplementary material.

Supplementary Material 1

## Data Availability

All data generated or analyzed during this study are included in the submitted article and its supplementary files.

## Abbreviations

CYP2E1: Cytochrome P450 2E1
CA: Cholic acid
HCC: Hepatocellular carcinoma
DEN: Diethylnitrosamine
TBA: Total bile acids
CDCA: Chenodesoxycholic acid
HBV: Hepatitis B virus
HCV: Hepatitis C virus
BAs: Bile acids
GCDC: Glycochenodeoxycholate
GGT: Gamma-glutamyltransferase
CCA: Cholangiocarcinoma carcinoma
SD: Sprague-Dawley
