# Supplementary material for: CYP2E1 deficit mediates cholic acid-induced malignant growth in hepatocellular carcinoma cells
Source: Mol Med. 2024 Jun 7;30:79. doi: 10.1186/s10020-024-00844-5 (PMC11157842; doi:10.1186/s10020-024-00844-5)
Supplement: Supplementary file 1 — Supplementary Material 1 [file 10020_2024_844_MOESM1_ESM.docx]

**Supplementary figures**

**Figure S1.** Differential proteins and biological pathways identified in tumor tissues of SD rats in the group DEN+CA and the group DEN. (A) Pathway enrichment analysis of differentially expressed proteins from the liver tumors specimens of male SD rats in group DEN+CA compared with the group DEN with Metascape online analysis (the significantly enriched pathway has been defined as *p* < 0.05). (B) Detected PPI modules and quantitative analysis of related differentially expressed proteins in the group DEN+CA compare with the group DEN.

**Figure S2.** AKT inhibition inverses CA-induced autophagy regulation and promotion growth of HCC cells. HepG2 and Huh7 cells were treated with CA supplemented with or without MK2206 for 24h. (A-B) The level of p62 was detected by western blot analysis (A) and quantitatively analyzed (B). (C-D) The representative images of colony formation were shown (C) and then quantitatively analyzed (D).
